# Recombinant BCG Expressing *Mycobacterium ulcerans* Ag85A Imparts Enhanced Protection against Experimental Buruli ulcer

**DOI:** 10.1371/journal.pntd.0004046

**Published:** 2015-09-22

**Authors:** Bryan E. Hart, Laura P. Hale, Sunhee Lee

**Affiliations:** 1 Human Vaccine Institute and Department of Medicine, Duke University Medical Center, Durham, North Carolina, United States of America; 2 Department of Pathology, Duke University, Durham, North Carolina, United States of America; Fondation Raoul Follereau, FRANCE

## Abstract

Buruli ulcer, an emerging tropical disease caused by *Mycobacterium ulcerans* (MU), is characterized by disfiguring skin necrosis and high morbidity. Relatively little is understood about the mode of transmission, pathogenesis, or host immune responses to MU infection. Due to significant reduction in quality of life for patients with extensive tissue scarring, and that a disproportionately high percentage of those affected are disadvantaged children, a Buruli ulcer vaccine would be greatly beneficial to the worldwide community. Previous studies have shown that mice inoculated with either *M*. *bovis* bacille Calmette–Guérin (BCG) or a DNA vaccine encoding the *M*. *ulcerans* mycolyl transferase, Ag85A (MU-Ag85A), are transiently protected against pathology caused by intradermal challenge with MU. Building upon this principle, we have generated quality-controlled, live-recombinant strains of BCG and *M*. *smegmatis* which express the immunodominant MU Ag85A. Priming with rBCG MU-Ag85A followed by an *M*. *smegmatis* MU-Ag85A boost strongly induced murine antigen-specific CD4^+^ T cells and elicited functional IFNγ-producing splenocytes which recognized MU-Ag85A peptide and whole *M*. *ulcerans* better than a BCG prime-boost vaccination. Strikingly, mice vaccinated with a single subcutaneous dose of BCG MU-Ag85A or prime-boost displayed significantly enhanced survival, reduced tissue pathology, and lower bacterial load compared to mice vaccinated with BCG. Importantly, this level of superior protection against experimental Buruli ulcer compared to BCG has not previously been achieved. These results suggest that use of BCG as a recombinant vehicle expressing MU antigens represents an effective Buruli ulcer vaccine strategy and warrants further antigen discovery to improve vaccine efficacy.

## Author Summary

Buruli ulcer, caused by subcutaneous infection with *Mycobacterium ulcerans*, is a highly disfiguring flesh-eating skin disease with significant morbidity. Besides surgical intervention, 8-week combination antibiotics is the standard of care. However, problems with resistance and toxicity warrant their replacement with efficacious vaccines. Several attempts to generate a vaccine have met with limited success and, to date, BCG remains the only vaccine capable of conferring transient protection. Here we demonstrate that a recombinant BCG-based vaccine expressing the immunodominant *M*. *ulcerans* Ag85A is capable of significantly enhancing protection in experimental Buruli ulcer compared to standard BCG, with a decrease in bacterial burden, pathology, and increase in survival. These results support further Buruli ulcer vaccine development using the highly safe and well-established BCG vehicle.

## Introduction

Buruli ulcer (BU), the clinical manifestation of subcutaneous infection by *Mycobacterium ulcerans* (MU), is a highly disfiguring flesh-eating skin disease with significant associated morbidity [1–5]. Much of the pathology related to *M*. *ulcerans* infection is due to secretion of the potent cytotoxin, mycolactone, which not only leads to tissue necrosis but also possess immunosuppressive and analgesic properties [6–8]. Although infections are present worldwide, endemicity occurs in impoverished areas with below-average access to appropriate diagnosis and medical treatment. Unfortunately this condition disproportionately afflicts children, and over 50% of those affected are less than 15 years of age [9]. Not only is Buruli ulcer capable of causing body-wide scarring, loss of limbs, damage to eyes, osteomyelitis, and secondary colonization of necrotic tissues, but infection is often affiliated with severely negative social stigma and ostracism [10,11]. If diagnosed early enough, an eight-week course of multiple anti-mycobacterial drugs are required to eliminate infection [12]. However, due to the typically indolent and misleading nature of initial disease symptoms, skin ulceration is often allowed to progress to a point at which antibiotic treatment is ineffective in preventing further necrosis. At this stage, surgical removal of infected tissue and skin grafting is required. The mode of transmission, pathogenesis, and host immune responses to *M*. *ulcerans* infection are poorly understood, and there is currently no effective prophylactic vaccine available [13].

*Mycobacterium bovis* bacille Calmette-Géurin (BCG) is the most widely administered vaccine in the world and is currently the only WHO-approved vaccine for tuberculosis [14–16]. Although BCG confers highly variable protection against pulmonary tuberculosis (TB), defense against disseminated forms of tuberculosis in children has been more consistently observed [17]. Previous retrospective studies have attempted to assess if vaccination with BCG offers protection against Buruli ulcer. Prior vaccination with BCG was observed to offer short term protection, mainly in the form of a delay in onset of tissue ulceration, although up to 45% protection was observed against MU-associated osteomyelitis [18–21]. Mouse models of Buruli ulcer infection have also examined the protective qualities of BCG vaccination and have yielded similar results, whereby the time to onset of footpad swelling in BCG-primed animals is delayed compared to unprimed controls [22].

Alternative anti-Buruli ulcer vaccination strategies have involved use of DNA-based vaccines which direct the expression of MU antigens within host cells. Roupie *et al*. utilized a plasmid-based vaccine to encode several mycolactone polyketide synthase domains, where DNA-primed mice were boosted with recombinant homologous proteins and were subsequently infected with virulent *M*. *ulcerans* 1615 [23]. Despite the evidence for antigen-specific antibody and T cell responses to this vaccination, little protection was observed and none of the vaccines yielded protection matching the level conferred by BCG priming. However in other studies, Tanghe *et al*. repeatedly observed that upon priming with two doses of plasmid DNA encoding MU mycolyl transferase Ag85A (MU-Ag85A) and subsequently boosting with purified recombinant protein, the onset of mouse footpad swelling was delayed to a level approaching that of BCG priming alone [24,25].

Based on the combined observations that both BCG inoculation and exposure to certain MU antigens could impart protection in the mouse model of *M*. *ulcerans* infection, we examined whether enhanced protection could be conferred by a prime of recombinant BCG (rBCG) strain overexpressing MU-Ag85A. Due to the fact that BCG was previously shown to be ineffective at boosting its own prime responses, we also examined the use of recombinant *M*. *smegmatis* expressing MU-Ag85A as a boosting agent [26]. Previous studies have highlighted the importance of T_h_1-based CD4^+^T cell responses during mycobacterial infection, and the skewing of adaptive immunity away from such responses have been observed to coincide with ulceration in Buruli ulcer patients [27–33]. In addition to the strong proliferation of CD4^+^ Ag85A-specific T cells and increase in functional IFNγ-producing splenocytes stimulated by our recombinant vaccine, MU bacterial load in the mouse footpad was significantly reduced. Most importantly, either a single administration of BCG-MUAg85A or the prime-boost model using recombinant mycobacteria significantly increased the time to onset of footpad swelling in MU-infected mice, demonstrating the first Buruli ulcer vaccine to achieve enhanced protection over BCG vaccination.

## Methods

### Mice

Female C57BL/6 mice were obtained from Jackson Laboratories. Mice were 6–8 weeks old at time of vaccination and 16–18 weeks old by time of challenge.

### Bacterial strains and culture

*Mycobacterium bovis* BCG-Danish (BCGD) was cultured in liquid Difco Middlebrook 7H9 media or on solid Difco Middlebrook 7H10 agar supplemented with 0.5% glycerol, OADC, and 0.05% tyloxapol. Selection of BCG transformants was accomplished by supplementing liquid or solid media with 25 or 40 μg/ml kanamycin, respectively. Liquid cultures less than 50 ml were grown at 37°C and shaken at 120 rpm. Liquid cultures greater than 50 ml were expanded to no more than 250 ml in one liter roller bottles and rotated at 6 rpm. Large-volume accession lots of vaccine strains were frozen in 1 ml aliquots at a concentration of OD_600_ 1 (~10^8^ CFU/ml). *Mycobacterium ulcerans* strain 1615 was kindly provided by Dr. Pamela Small (University of Tennessee) and was grown in Middlebrook 7H9 or 7H10 as above at 32°C. For sequencing and manipulation of plasmid DNA, DH5α *Escherichia coli* (*E*. *coli*)was grown in lysogeny broth (LB) or on agar plates supplemented with appropriate antibiotics.

### Plasmid constructions and transformation

The *M*. *ulcerans* Ag85A open reading frame with its endogenous signal sequence was amplified from genomic DNA using the following primers engineered with NdeI and EcoRV restriction sites: forward, 5’-GAGACATATGAAGCTTGTTGACAGGTTTCGTGGC-3’, reverse, 5’-GAGAGATATCGGCGCCCTGGG TGTCACC-3.’ MU-Ag85A amplicons were cloned into the pMV261 backbone, where antigen expression was under the control of the constitutive mycobacterial *hsp60* promoter and plasmid selection was mediated by kanamycin resistance (henceforth, pSL401). The hemagglutinin (HA) epitope was fused to the C-terminus of MU-Ag85A. Electrocompetent BCG and *M*. *smegmatis* cells were prepared by pelleting log phase liquid cultures (OD_600_ 0.6–0.8) at 3000 rpm for 10 minutes and washing three times in 10% glycerol with 0.05% tyloxopol. Mycobacteria were electroporated using 0.5 μg plasmid DNA and recovered in Middlebrook 7H9 media at 37°C overnight.

### Vaccine lot quality control

Accession lot of vaccine strains underwent quality control to assess antigen expression, absence of contamination, and retention of plasmid DNA. For immunoblotting, bacterial lysates were prepared by pelleting 10 ml of log-phase culture at 3000 rpm for five minutes. Pellets were washed by repeated centrifugation and resuspension in 10 ml PBST. The final pellet was resuspended in 200 μl lysis buffer with glass beads and vortexed for three minutes. SDS PAGE gels were loaded with a mixture of 15 μl clarified lysate with Laemmli buffer and run under 130V for one hour. Protein was subsequently transferred to PVDF membranes by electrophoresis under 30V for one hour. Membranes were blocked by shaking in 5% milk dissolved in TBS with 0.1% tween (TBST) at room temperature for one hour. HRP-conjugated mouse anti-HA (clone 3F10, Roche) antibodies were diluted 1:1000 in 5% milk-TBST and incubated with membranes for one hour at room temperature. After washing, proteins were detected via chemiluminescence (Lumi-light, Roche) and exposure of X-ray film. For plasmid sequencing, plasmid isolation was performed by incubating mycobacterial pellets with Qiagen Miniprep buffer P1 and lysozyme at 60°C for one hour. This was followed by the manufacturer’s protocol. Eluted plasmid DNA was then heat shocked into chemically competent *E*. *coli* DH5α and plasmid DNA was isolated from the resulting transformant colonies. The presence of the correct plasmid insert was assessed by gel electrophoresis of NdeI/EcoRV restriction digests. Plasmid inserts were also sequenced and analyzed using Clone Manager (Sci-Ed) software. For the assessment of contamination 100 μl of thawed accession lot material was spread plated on chocolate agar and incubated at 37°C for up to two weeks.

### Mouse vaccination and infection

For priming, C57BL/6 mice were vaccinated subcutaneously in the scruff of the neck with 100 μl (~10^7^ cells) from a thawed vaccine accession lot vial of BCG-empty vector or recombinant BCG-MU-Ag85A. Eight weeks post-prime, mice were boosted retro-orbitally with 100 μl (~10^7^ cells) of *M*. *smegmatis*-MU-Ag85A and were then challenged intradermally via the footpad with 10^5^ *M*. *ulcerans* 1615 at two weeks post-boost. Vials of MU1615 challenge material were consistently pulled from the same accession lot and were fully virulent by testing pathology in mouse footpad models. At two to three week intervals, the height and width of footpads from infected mice were measured with digital calipers for signs of swelling. To comply with IACUC protocol, mice were euthanized once height of swelling exceeded 4.5 mm (prior to visible ulceration), in order to prevent animal suffering.

### Ag85-MHCII tetramer assay

Levels of Ag85-specific CD4+ T cells were assessed by tetramer staining and flow cytometric analysis. At weekly time points after BCG inoculation, blood samples were collected retro-orbitally and peripheral blood mononuclear cells (PBMCs) were isolated by gradient centrifugation through 1 ml of Lympholyte M (Cedarlane). The resulting PBMC layers were removed, washed in 10 ml PBS, and were resuspended in 2 ml of ACK lysis buffer for three minutes to eliminate erythrocyte contamination. PBMC pellets were then washed and resuspended in APC-conjugated *M*. *tuberculosis* Ag85B-MHCII tetramer (1:500, NIH Tetramer Core Facility) diluted in flow buffer (2% FBS in PBS). This tetramer recognizes a 15 amino acid epitope (FQDAYNAAGGHNAVF) with high sequence homology to *M*. *ulcerans* Ag85A. The staining proceeded for 30 minutes at 37°C followed by addition of 1:500 FITC-conjugated anti-mouse CD4 (clone GK-1.5, Biolegend) and 1:200 PE-Cy5 anti-mouse CD8 (clone 53–6.7, Biolegend) for 30 minutes on ice. The PBMCs were subsequently washed in 3 ml of flow buffer, followed by resuspension in 4% paraformaldehyde. Samples were fixed for 30 minutes before flow cytometric analysis using a BD LSRII and FlowJo analysis software (Tree Star Inc.). For central and effector memory cell staining, the above tetramer protocol was combined with additional antibody incubations, including 30 minutes on ice using APC-Cy7 anti-mouse CD4 (clone GK-1.5), PE-Cy7 anti-mouse CD44 (clone IM7, BD Pharmingen), and FITC anti-mouseCD62L (clone MEL-14, BD Pharmingen).

### Acid fast bacilli microscopy

At weeks 5 and 12 post-challenge, mice were euthanized and infected footpads were removed for disinfection and homogenization. Footpads were disinfected by submerging in 70% ethanol for five minutes and subsequently washing three times with PBST. Footpads were then were finely minced using surgical scissors and placed in mortar and pestles for homogenization. These homogenates were then subjected to N-acetyl-L-cysteine (NALC)/NaOH disinfection by combining with a 50:50 mixture of 4% NaOH and 2.9% sodium citrate containing 1% NALC. Disinfection was allowed to proceed for 20 minutes at room temperature. Homogenates were then pelleted at 3000 rpm for 5 minutes, washed three times in PBST, and were filtered through a 40 μm mesh. Glass microscope slides were marked with a 0.79 cm^2^ circle and 5 μl of filtrate was spread evenly within this area. After drying the smear, slides were heat-fixed by passing briefly over a Bunsen burner flame and then were allowed to cool. Smears were stained using auramine-rhodamine (BD Bioscienes) for three minutes, followed by a rinsing in water, destaining using acid alcohol, counterstaining with potassium permanganate, and rinsing again. Dried slides were viewed at 100x oil immersion under a Nikon X. Acid-fast bacilli (AFB) were enumerated within four random fields of view (FOV) per animal (16 images total per vaccinated group) and total AFB were calculated by multiplying counts by the numbers of 0.038 mm^2^ FOVs under the 100x lens per marked smear area per microliter of filtrate applied.

### IFNγ ELISPOT

96-well PVDF plates were equilibrated with 70% ethanol, washed with PBS, and coated with 1 μg/ml capture anti-mouse IFNγ antibody (clone AN18, Mabtech) overnight. Mice that had received the full vaccination scheme described above were euthanized two weeks after boost to isolate splenocytes in RPMI complete (RPMI with L-glutamine and 10% fetal bovine serum). After blocking plates with RPMI media, 6 x 10^5^ splenocytes were added to each well along with various agonists: ConA positive control (100 μg/ml), MU-Ag85A peptide (100 μg/ml), or heat killed *M*. *ulcerans* (1 mg/ml). Following a 16 hour stimulation at 37°C, the plates were washed with PBS + 0.05% tween 20 and 1:1000 secondary anti- mouse IFNγ antibody (clone R46A2, Mabtech) was added for two hours at 37°C. The plates were washed again before addition of VectaStain avidin peroxidase complex (Vector Labs) for one hour at room temperature. 3-amino-9-ethylcarbazole substrate in acetate buffer was added for five minutes until the reaction was stopped by submerging plates in deionized water. Plates were dried overnight and spots were enumerated using a CTL Immunospot plate reader.

### Ethics statement

This study was reviewed and approved by the Duke University Institutional Animal Care and Use Committee (IACUCU protocol A065-13-03). Duke IACUC protocols adhere to the USDA, AAALAC, Animal Welfare Act, Guide for Care and Use of Laboratory Animals and Public Health Service Policy on Humane Care and Use of Laboratory Animals.

## Results

### Stable expression of *M*. *ulcerans* Ag85A in recombinant BCG and *M*. *smegmatis*

**Fig 1:**
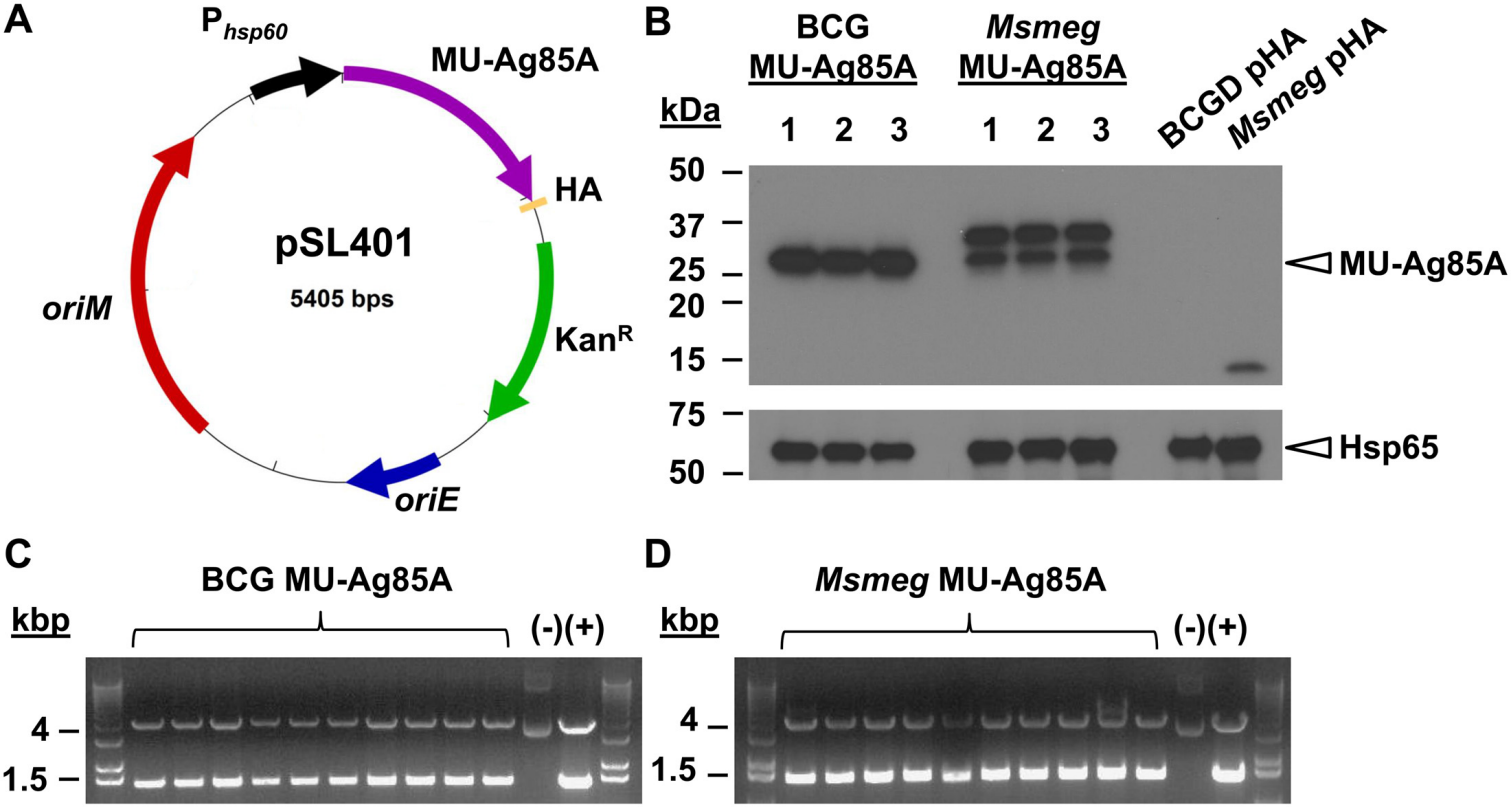
Quality-controlled, heterologous expression of MU-Ag85A by BCG and *M*. *smegmatis*. **A.** The plasmid map for pSL401 is shown. Constitutive expression of MU-Ag85A (purple) fused to an HA epitope tag (yellow) is driven by the promoter region from *hsp60* (P_*hsp60*_, black). Plasmid replication is regulated in *E*. *coli* using *oriE* (blue) and in mycobacteria using *oriM* (red). Plasmid retention is selected by the presence of a kanamycin resistance cassette (hashed) **B.** Three vaccine accession lot vials of BCG or *M*. *smegmatis* (*Msmeg*) transformed with pSL401 were chosen at random for Western analysis of MU-Ag85A expression using anti-HA antibody. The expected band size was 35 kDa. Negative controls are lysates from BCG or *Msmeg* transformed with empty vector (BCG pHA, *Msmeg* pHA). Loading controls were performed by detection of Hsp65. Plasmid DNA was isolated from BCG (C) and *Msmeg* (D) vaccine lots and transformed into chemically competent *E*. *coli*. 10 transformants were plasmid prepped and restriction digested (*DraI/NheI*) to assess presence of MU-Ag85A insert and correct band size (1.4 kb). Undigested and digested pSL401 plasmids were run as negative and positive controls, respectively.

In order to express the immunodominant Ag85A from *M*. *ulcerans* (MU-Ag85A) in recombinant mycobacterial vectors, *M*. *bovis* BCG (BCG) and *M*. *smegmatis* (*Msmeg*), electrocompetent cells were transformed with pSL401 (Fig 1A). This replicating plasmid drives transcription of MU-Ag85A containing a C-terminal fusion to hemagglutinin (HA) using the strong, constitutive *hsp60* promoter. Mycobacterial transformants were selected by plasmid-encoded resistance to kanamycin and replication of plasmids was regulated by *oriM* in mycobacteria, and by *oriE* in *E*. *coli* shuttle strains. Large-volume vaccine accession lots of transformants were frozen and a quality control protocol was used to assess antigen expression, vaccine lot purity, and plasmid sequence integrity.

First, three random vials of each lot were thawed for Western analysis of MU-Ag85A expression using anti-HA antibody. Fig 1B displays the expected 35 kDa molecular weight band for MU-Ag85A expressed in BCG (BCG MU-Ag85A). Expression of the antigen in *Msmeg* appears to generate an additional larger band, which was not a result of plasmid mutation upon sequencing. Accession lots of BCG and *Msmeg* transformed with empty-vector DNA, BCG pHA and *Msmeg* pHA, were also generated to be used as negative controls in subsequent studies. As expected, lysates from these lots did not yield anti-HA reactive bands on the same immunoblot.

Due to potential instability of recombinant inserts when expressed in mycobacteria, an analysis of plasmid integrity was performed on both the BCG MU-Ag85A and *Msmeg* MU-Ag85A lots. Re-isolated pSL401 plasmid DNA from three thawed accession lots vials was examined for appropriately sized MU-Ag85A inserts and sequence. Ten out of ten plasmid transformants from each vaccine lot contained the correct MU-Ag85A insert band, suggesting a high proportion of recombinant mycobacteria retained the plasmid (Fig 1C and 1D). Sanger-sequencing of these re-isolated plasmids was performed to ensure all transformants also contained the correct MU-Ag85A sequence devoid of extraneous mutation.

A final aspect of the quality control procedure was to identify any contaminating microorganisms present in the accession lots which could confound subsequent studies. No contamination was observed after incubating thawed accession lot material on both chocolate agar and mycobacterial growth medium for several weeks. Upon establishing the integrity of stable antigen expression and both plasmid and culture purity, BCG MU-Ag85A and *Msmeg* MU-Ag85A vaccine lots were ready for further immunological and protection studies.

### Proliferation of antigen-specific CD4^+^ T cells following vaccination with BCG MU-Ag85A

**Fig 2:**
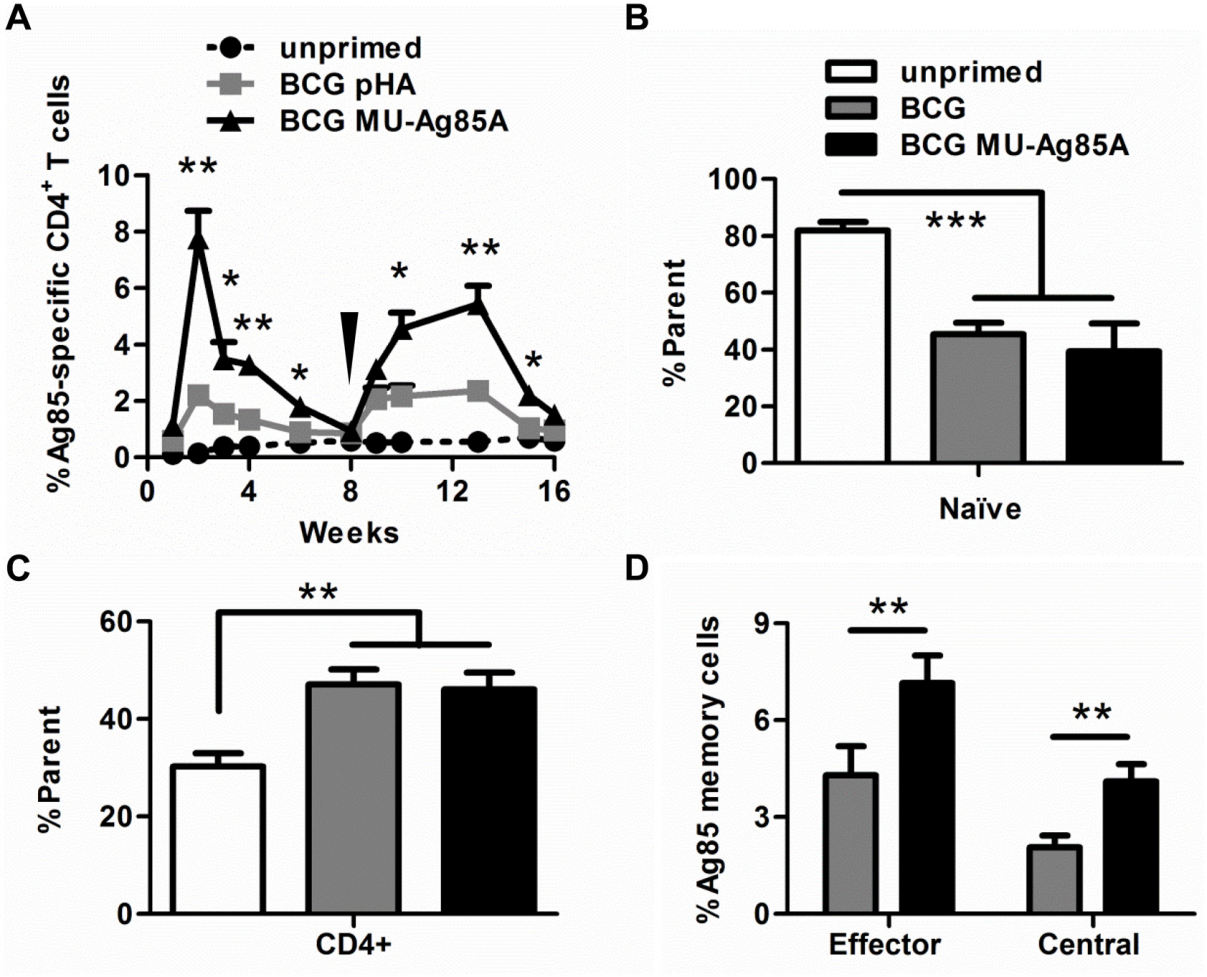
BCG MU-Ag85A vaccination induces proliferation of antigen-specific CD4+ T cells and immunological memory. **A.** C57BL/6 mice were left unprimed (dotted black) or were intravenously primed using 10^7^ BCG transformed with empty vector (BCG pHA, gray) or with pSL401 (BCG MU-Ag85A, black). At various time points, peripheral submandibular blood was collected. T lymphocytes were isolated by centrifugation and flow cytometric analysis was performed to quantify levels of CD4^+^ T cells bound to MHCII-Ag85 tetramer. At 8 weeks post-prime (black arrow), mice were intravenously boosted with 10^7^ *Msmeg* expressing MU-Ag85A. Blood was similarly collected and analyzed for percent MU-Ag85A-specific CD4^+^ T cells. Asterisks indicate statistical analysis by the student’s t-test (n = 5 for each group) comparing the BCG pHA to BCG MU-Ag85A groups. Error bars represent standard deviation. *p<0.05, **p<0.005 **B-D.** At 3 weeks post-prime, mice were bled retro-orbitally and peripheral CD4^+^ T cells were stained for memory markers. Percentages of total CD62L_lo_CD44_lo_ naïve (B) and CD4+ T cells (C) were quantified, as were effector memory T cells (CD62L_lo_CD44_hi_) and central memory T cells (CD62L_hi_CD44_hi_) which specifically bound Ag85-MHCII-tetramer (D). Asterisks indicate statistical analysis by the student’s t-test (n = 4 for each group). Error bars represent standard deviation. **p<0.005, ***p<0.001

The production of CD4^+^ T cell-mediated responses is vital for anti-mycobacterial immunity [31,32]. To determine if vaccination with BCG MU-Ag85A could generate an antigen-specific adaptive immune response, C57BL/6 mice were primed with 10^7^ bacilli by intravenous injection. At intervals ranging from 1–8 weeks post-prime, peripheral blood samples were collected for isolation of circulating lymphocytes. Flow cytometric analysis of MHCII tetramer staining was used to identify the percentage of CD4^+^ T cells which recognized Ag85A. As seen in Fig 2A, BCG MU-Ag85A induced significantly larger populations of Ag85A-specific helper T cells compared to BCG-pHA vaccinated or unprimed mice. Background tetramer-positive T cell populations were most likely observed during BCG pHA priming due to endogenous Ag85A expressed by BCG.

Over the course of initial BCG prime, populations of Ag85A-specific T cells peaked at 2 weeks and waned to pre-vaccination levels by week 8. Previous reports have determined that multiple doses of BCG are not effective at boosting primary responses in humans [26]. In order to determine if a heterologous mycobacterium could be used to boost initial T cell proliferation, an intravenous dose of 10^7^ *Msmeg* MU-Ag85A was administered at 8 weeks post-prime. One week post-boost, circulating Ag85A-specific T cells increased over 3-fold and remained significantly higher compared to BCG-primed mice. Additionally, maximal levels of Ag85A-specific T cells were maintained for 3-fold longer following the boost than after priming.

Establishment of memory CD4^+^ T cell reservoirs has been shown to be important for development and downstream efficacy of anti-mycobacterial vaccines [34,35]. To examine the level of antigen-specific memory cells induced by vaccination with BCG MU-Ag85A, C57BL/6 mice were primed with 10^7^ bacilli retro-orbitally. Three weeks later, peripheral lymphocytes were stained for CD4 and the T cell memory markers, CD62L and CD44. Priming alone with either BCG pHA or BCG MU-Ag85A halved the naïve (CD62L_lo_CD44_lo_) population of cells (Fig 2B) while increasing the total population of CD4^+^ T cells by 20% (Fig 2C). As predicted by the Ag85 MHCII tetramer experiment in Fig 2A, the levels of antigen-specific memory cells were significantly higher in BCG MU-Ag85A vaccinated mice, with both effector and central CD4^+^ memory populations increasing by 1.7 and 2-fold respectively. Together these data suggested that utilization of BCG MU-Ag85A as a vaccine prime or *Msmeg* MU-Ag85A as a boost is an effective regimen for producing high levels of CD4^+^ helper T cells and memory populations capable of recognizing an immunogenic MU protein.

### Expression of MU-Ag85A increased responsiveness of T_h_1 cells to *M*. *ulcerans* antigens

Many studies have highlighted the requirement of T_h_1 IFNγ-producing responses for inducing successful anti-mycobacterial immunity (27–33). In order to determine if the antigen-specific T cells produced during vaccination could generate these types of responses, C57BL/6 mice were intravenously primed with recombinant BCG MU-Ag85A for 8 weeks, then boosted with *Msmeg* MU-Ag85A. As a control, a group of mice was also primed with *Msmeg* MU-Ag85A, and then homologously boosted with the same strain. Two weeks post-boost, the mice were euthanized and splenocytes were harvested for *in vitro* stimulation with either MU-Ag85A peptide or heat-killed *M*. *ulcerans* 1615 (HKMU).

**Fig 3:**
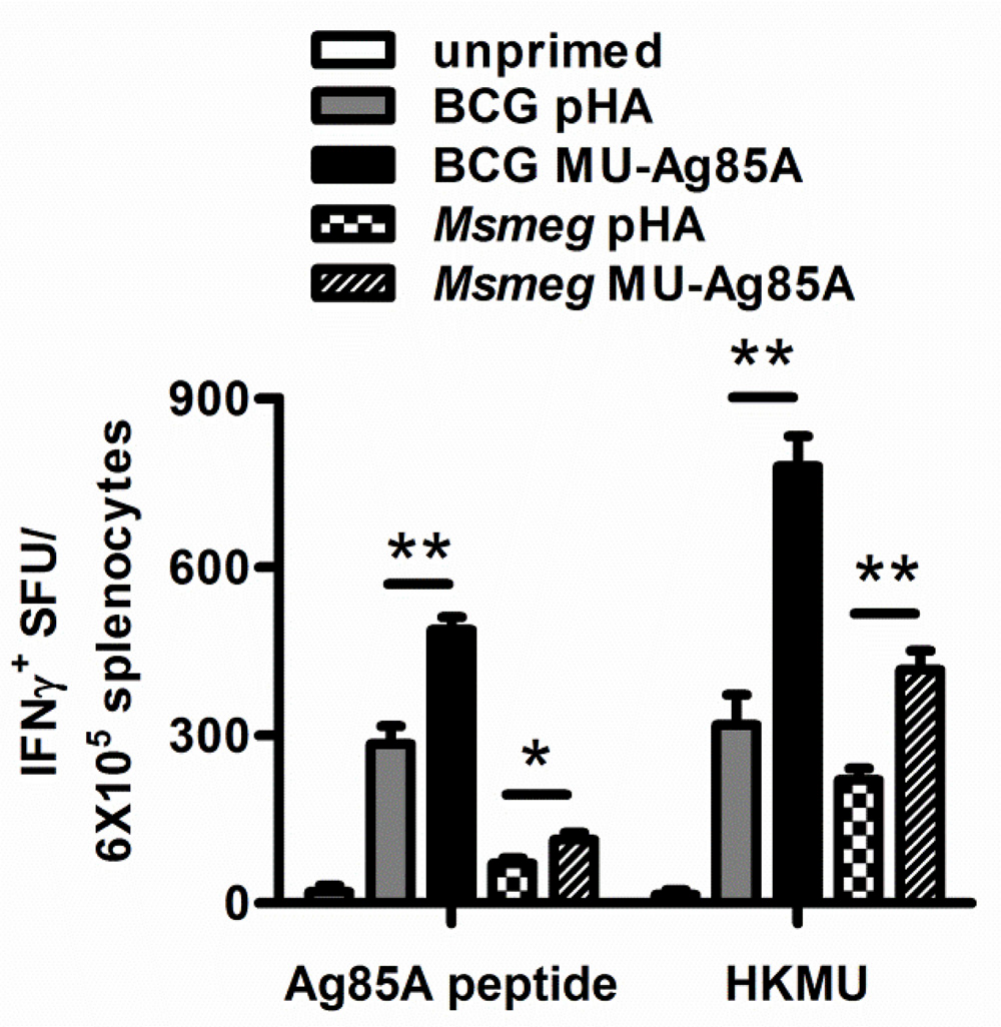
Expression of MU-Ag85A increases responsiveness of T_h_1 cells to *M*. *ulcerans* antigens. C57BL/6 mice were left unprimed (white) or were intravenously primed with 10^7^ BCG transformed with empty vector (BCG pHA, gray), *Msmeg* pHA (checkered), BCG MU-Ag85A (black), or *Msmeg* MU-Ag85A (hashed). At 8 weeks post-prime, mice were intravenously boosted with 10^7^ *Msmeg* expressing MU-Ag85A. Two weeks following the boost, mice were euthanized and splenocytes were isolated for stimulation with MU-Ag85A peptide or heat killed *M*. *ulcerans* 1615 (HKMU). ELISPOT spot forming units (SFU) were used to determine number of IFNγ-producing splenocytes after 24 hours of stimulation. Asterisks indicate statistical analysis by the student’s t-test (n = 5 for each group). Error bars represent standard deviation. *p<0.05, **p<0.005

Enzyme-linked immunospot (ELISPOT) was then used to quantify the number of functional T_h_1 splenocytes capable of producing IFNγ in response to MU antigens. Fig 3 displays the number of IFNγ^+^ spot-forming units (SFU) counted 24 hours after agonist stimulation. Both BCG pHA and *Msmeg* pHA vaccinations were capable of generating responses, however, priming with BCG MU-Ag85A yielded significantly greater numbers of IFNγ^+^ splenocytes. The strongest responses were observed when splenocytes were stimulated with HKMU, against which priming with BCG MU-Ag85A increased SFU over BCG pHA by 2.5-fold. Similar trends were observed using *Msmeg* MU-Ag85A as a prime, although overall responses were much lower than those observed using BCG strains. These data suggested that expression of MU-Ag85A in the BCG background increases responsiveness of functional T_h_1 cells not only to Ag85A, but also to other antigens contained within *M*. *ulcerans* bacteria.

### Superior protective effects of subcutaneous vaccination with BCG MU-Ag85A

**Fig 4:**
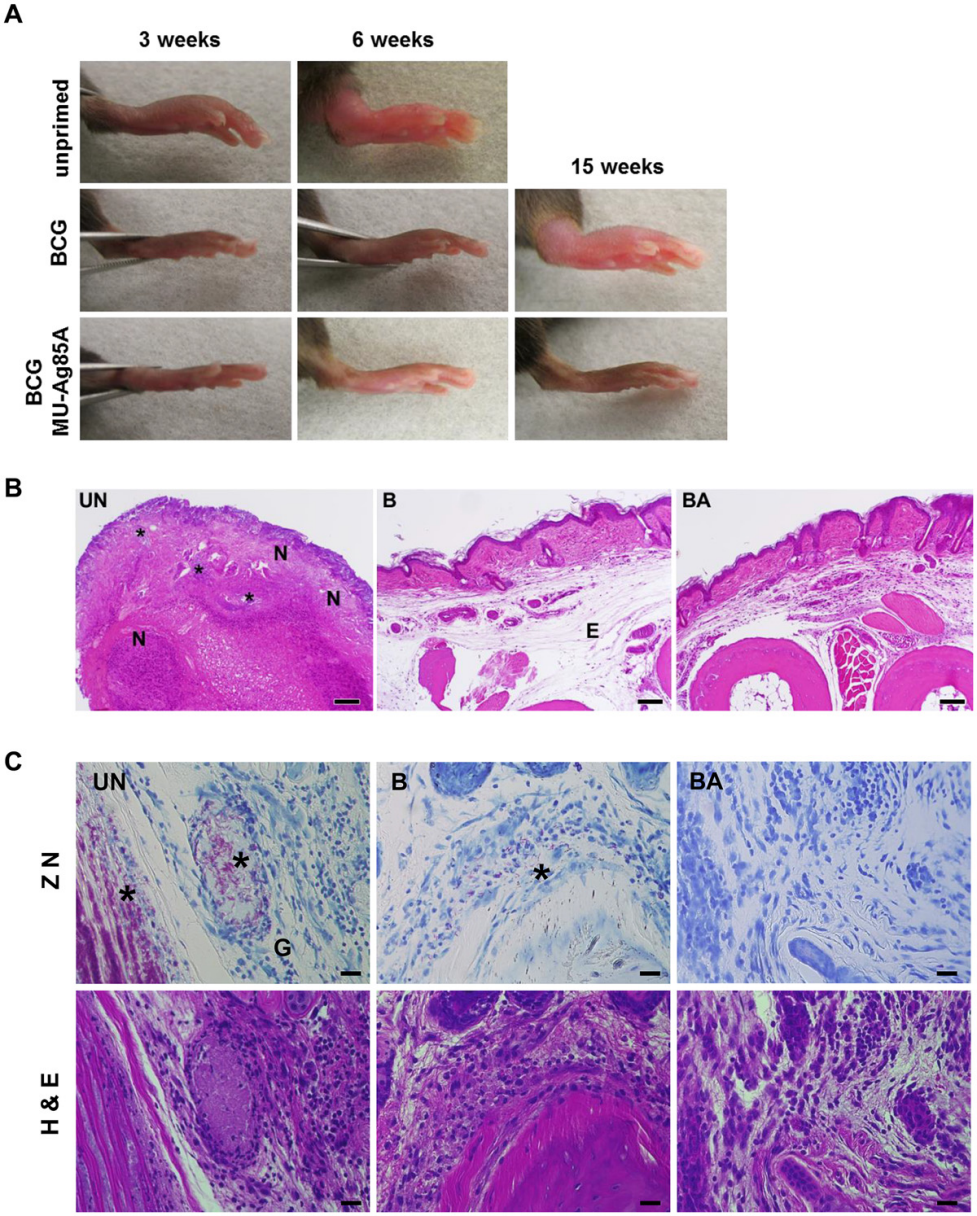
Footpad swelling and tissue pathology are reduced upon vaccination with BCG MU-Ag85A. C57BL/6 mice were left unprimed or were subcutaneously primed with 10^7^ BCG transformed with empty vector (BCG pHA), or BCG MU-Ag85A. At 8 weeks post-prime, mice were intravenously boosted with 10^7^ *Msmeg* expressing MU-Ag85A. Two weeks following the boost, mice were challenged with 10^5^ MU1615 intradermally via the left hind leg footpad. Shown are representative images of infected footpads at 3, 6, and 15 weeks post-challenge. Mice were euthanized if vertical swelling surpassed 4.5 mm, as was the case for unprimed mice before week 15. B. Representative H&E stained tissue sections from 12 week MU-infected mouse footpads are shown. Paws of unprimed mice (UN) consistently showed ulceration (loss of epidermis) with abundant necrosis (N) and infiltrates of inflammatory cells (*). Edema (E) remained prominent in BCG pHA-primed (B) mice but was rarely associated with BCG MU-Ag85A-primed (BA) mouse footpads. Scale bar, 100 μm. C. Representative Ziehl-Neelson (ZN, top row) and H&E (bottom row)-stained tissue sections from 12 week MU-infected mouse footpads are shown. Granulomatous lesions (G) and large masses of acid fast bacilli (AFB,*) are apparent in unprimed footpads (UN), while groups of extracellular AFB were detected in BCG-primed mice (B). AFB generally presented as single scattered extracellular bacteria in footpad tissues from BCG MU-Ag85A-primed mice (BA) and could not be detected in the footpad sections available for ZN staining. Scale bars indicate 100 μm.

Previous studies have shown that BCG vaccination in mice delays the onset of MU-induced pathology in the footpad model of Buruli ulcer [22,36]. To determine if vaccination with BCG MU-Ag85A could deliver improved control of MU infection compared to standard BCG vaccination, we carried out a direct comparison in the footpad challenge model. To recapitulate a more physiologically relevant vaccination route as used in humans, these groups of mice were subcutaneously primed. A 10^7^ subcutaneous does of recombinant mycobacteria was chosen because it was found to be superior over lower does in protection against MU challenge (S1 Fig). Following intradermal challenge with 10^5^ virulent MU1615, the height and width of challenged footpads were measured using digital calipers over the course of infection. After footpad vertical swelling reached 4.5 mm, mice were euthanized to eliminate suffering. In C57BL/6 mice footpad swelling reaches its peak after an average of 6–8 weeks post-challenge, although swelling can readily be observed by week 3 post-challenge (Fig 4A). Upon priming with BCG-pHA, the average peak swelling was reached at 12–15 weeks post-challenge, however, the use of BCG MU-Ag85A as a prime extended the time required for manifestation of peak swelling to 25–30 weeks post-challenge. In contrast, priming with *Msmeg* pHA afforded no protection against footpad swelling and the use of *Msmeg* MU-Ag85A only showed a slight trend toward delay in footpad swelling (S2 Fig).

Mycolactone production is known to contribute to the histological observations of necrosis within MU-infected tissues [37]. MU-infected footpads were collected for histopathological analysis to assess internal tissue damage in unprimed or vaccinated animals at 12 week post-challenge. Fig 4B displays representative images from H&E stained tissue sections from an unprimed, BCGD pHA-primed, or a BCG MUAg85A-primed mouse. In unprimed mice, footpads consistently showed ulcerative loss of epidermis with micro-hemorrhaging, as well as large areas of internal necrosis and general inflammatory infiltrate. These features were rare amongst BCGD MUAg85A-primed footpads, which was consistent with the reduced swelling observed at this time point. BCG pHA-primed footpads exhibited an intermediate phenotype, where prominent edema replaced necrotic features and was concordant with the level of swelling observed at week 12 post-challenge. To visualize and assess organization of *M*. *ulcerans in vivo*, Ziehl-Neelson staining was also performed on tissue sections from infected footpads of vaccinated or unvaccinated mice. Granulomatous lesions and large masses of extracellular acid fast bacilli (AFB) were observed in unprimed footpads, while groups of extracellular AFB were detected in BCG-primed mice (Fig 4C). However, AFB could not be detected in the BCG MU-Ag85A-primed footpad sections available for ZN staining suggesting a comparatively lower level of overall bacterial burden in these tissues.

The delay in footpad swelling and reduction in bacterial burden induced by BCG vaccination has previously been shown to temporarily protect mice against the need for euthanasia after MU challenge [22,36]. To determine if BCG MU-Ag85A could enhance the protective phenotype observed over BCG vaccination, C57BL/6 mice were subcutaneously primed with BCG or recombinant BCG alone, or BCG- primed and heterologously boosted with *Msmeg* MU-Ag85A as before. Ten weeks later, mice were challenged with an intradermal footpad injection of high dose (10^5^ bacilli) virulent MU 1615. Once infection induced vertical footpad swelling surpassing 4.5 mm, mice were euthanized.

**Fig 5:**
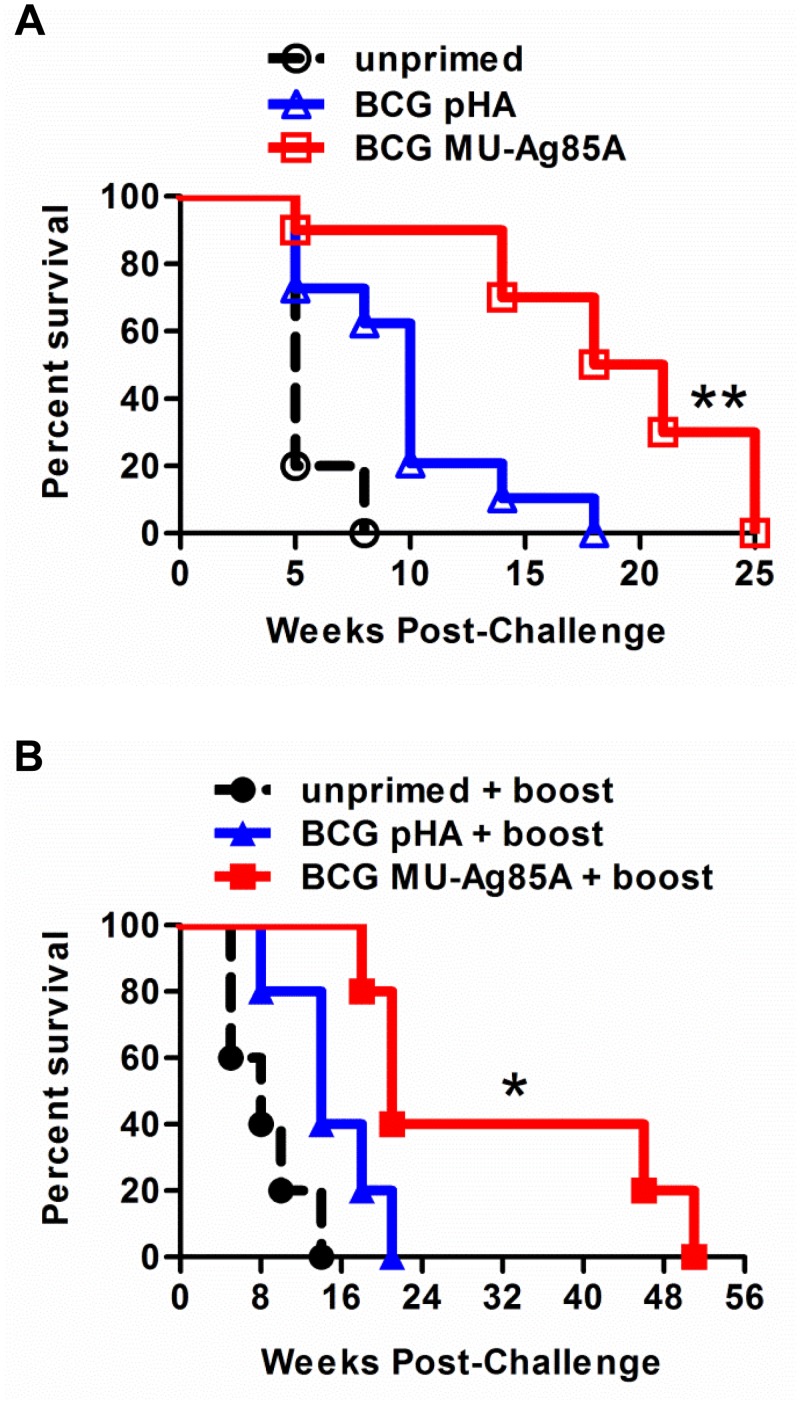
Superior protective effects of subcutaneous vaccination with BCG MU-Ag85A. **A.** Naïve C57BL/6 mice (dotted black) or mice subcutaneously primed with empty-vector BCG pHA (gray) or BCG MU-Ag85A (black) were challenged with 10^5^ MU1615 via the footpad ten weeks post-vaccination. Area of footpad swelling was measured at various time points post-challenge. Mice were euthanized if vertical footpad swelling surpassed 4.5 mm and survival represents time to euthanasia. Asterisk indicates statistical analysis of BCG pHA versus BCG MU-Ag85A by the Mantel-Cox test (n = 10 for each group). **B.** Survival was assessed in MU1615-challenged mice which had been subcutaneously primed with BCG pHA (gray) or BCG MU-Ag85A (black) for eight weeks, followed by a two week boost with *Msmeg* MU-Ag85A. Asterisk indicates statistical analysis of BCG pHA versus BCG MU-Ag85A by the Mantel-Cox test (n = 10 for each group). *p<0.03, **p<0.002

Fig 5A displays survival curves (time-to-euthanasia) for mice left unprimed or primed with either BCG pHA or BCG MU-Ag85A. While priming with BCG pHA could significantly increase protection over unprimed mice (p = 0.002), strikingly, a single subcutaneous vaccination with BCG MU-Ag85A further significantly increased survival in mice challenged with virulent MU 1615 (p<0.0001). Importantly, compared to the BCG prime alone, use of BCG MU-Ag85A was significantly more efficacious against experimental Buruli ulcer in mice (p<0.002). Furthermore, boosting BCG MU-Ag85A with an injection of *Msmeg*-MUAg85A at week 8 post-prime appeared to enhance the protective efficacy of this vaccine, even when compared to unprimed mice or BCG pHA-primed mice that also received a boost (Fig 5B) (p<0.03). Interestingly, unprimed mice which received a dose of *Msmeg* MU-Ag85A did significantly increase protection over unboosted, unprimed mice to a small degree (p<0.05), however, administration of the *Msmeg* MU-Ag85A boost did not statistically significantly increase protection in BCG pHA or BCG MU-Ag85A-primed mice compared to primes which did not receive a boost (p<0.07 and p<0.1, respectively). Significant protection was not observed when the *Msmeg* background itself was used as a prime, regardless of recombinant insert or boosting (S2 Fig). Overall these data suggest that the anti-mycobacterial immune response generated by increased proliferation of functional and antigen-specific CD4^+^ T cells can contribute to decreased swelling, reduced bacterial burdens, and an overall greater lifespan for MU 1615 infected mice.

### Subcutaneous vaccination with BCG MU-Ag85A significantly reduced footpad bacterial load compared to BCG

**Fig 6:**
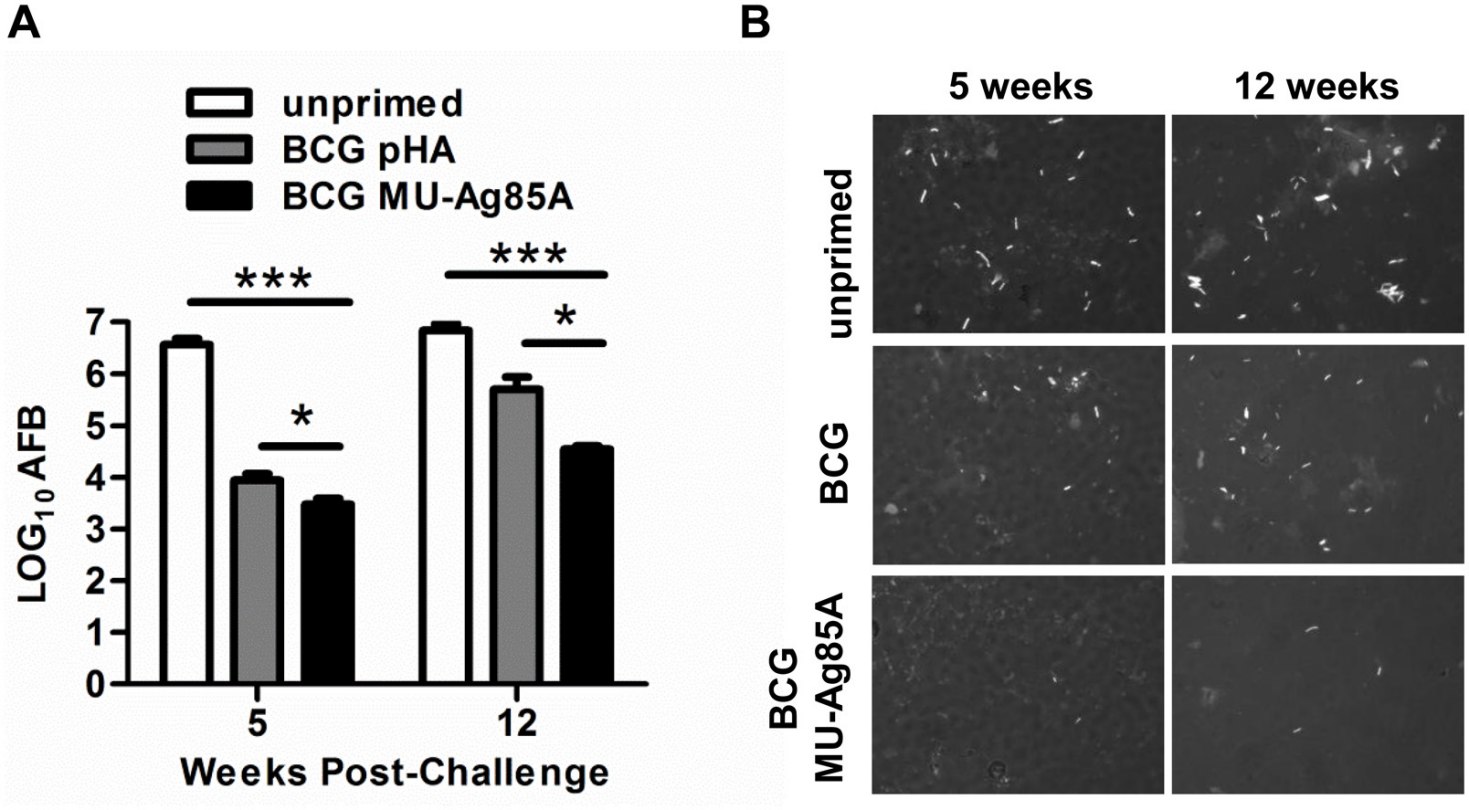
Vaccination with BCG MU-Ag85A significantly reduces footpad bacterial load at weeks 5 and 12 over BCG. **A.** C57BL/6 mice were subcutaneously vaccinated as previously described (unprimed; white, empty-vector BCG pHA; gray, BCG MU-Ag85A; black) and challenged with 10^5^ MU1615. At 5 and 12 weeks post-challenge, mice were euthanized and footpad homogenates were smeared on glass slides for auramine-rhodamine staining. Acid-fast bacilli (AFB) were quantified under 1000x magnification. Asterisks indicate statistical analysis by the student’s t-test (n = 16 images per group). Error bars represent standard deviation. ***p<0.001, *p<0.02 **B.** Shown are representative images from unprimed, BCG pHA, and BCG MU-Ag85A primed footpad smears at 5 and 12 weeks post-challenge.

Previous studies of experimental Buruli ulcer in mice have shown a correlation between the degree of footpad swelling and the *M*. *ulcerans* bacterial load in infected tissues [38]. To determine if the observed reduction in swelling and enhanced survival associated with BCG MU-Ag85A vaccination correlated with lower bacterial burdens, mice which had received vaccinations and challenges were euthanized for isolation of persistent MU 1615 in the challenged footpads. Infected footpads were removed from groups at 5 and 12 weeks post-challenge and acid-fast bacilli present in smears from filtered footpad homogenates were stained with auramine-rhodamine. Importantly, microscopic evaluation of bacterial load was assessed to ensure similar bacterial counts were achieved when compared to plating of colony forming units (CFU). As seen in S3 Fig, comparable trends were observed between acid fast staining of footpad homogenates and CFU plating. Fig 6A shows the average MU burdens for mice left unprimed or primed with BCG pHA or BCG MU-Ag85A during two time points post-challenge. Representative images of re-isolated MU can be seen in Fig 6B. At both 5 and 12 weeks post-challenge, BCG MU-Ag85A subcutaneous vaccination significantly reduced the footpad bacterial burden compared to unprimed mice by 3.1-log and 2.3-log, respectively. Importantly however, priming with BCG MU-Ag85A at both time points yielded significantly better protection against bacterial replication in the footpads compared to BCG pHA-primed mice, displaying a 0.5-log and 1.2-log reduction at 5 and 12 weeks respectively. This reduction in bacterial burden correlated well with the differential ability of each vaccine to reduce footpad swelling at similar time points post-infection (Fig 4A) and represents a potential mechanism for BCG MU-Ag85A-mediated protection against MU pathology.

## Discussion

Buruli ulcer is an insidious disease whose persistence and high morbidity is complicated by both social stigma of those infected and relatively poor access to diagnosis and healthcare in the most afflicted areas. The current standard of care requires lengthy adherence to rifampin and daily intramuscular streptomycin, drugs which are associated with side effects including nephrotoxicity and hearing loss [39]. This, added to the disproportionately high infection rates in children, makes it apparent that a Buruli ulcer vaccine is greatly needed. Previous studies in both humans and animal models have demonstrated that *Mycobacterium bovis* BCG vaccination affords some level of protection against the pathology observed during MU infection, although total protection has not been achieved. Interestingly, although exposure to MU antigens in the form of DNA vaccination or homologous protein boosting can readily induce antigen-specific T cell and antibody responses in mice, no vaccine has been able to achieve a level of protection better than BCG inoculation. Comparable to tuberculosis, the importance of T cells and T_h_1 responses to the containment of MU during infection has been established, but the necessary sub-populations, antigens, and cytokine milieu which correlate with protection against Buruli ulcer have not been fully recognized.

However, the immune responses and protection developed by exposure to the immunodominant *M*. *tuberculosis* mycolyl transferase, Ag85A, in mice and humans have been well characterized [40–42]. Previous studies have shown that DNA-based or rBCG vaccines encoding TB-Ag85A can generate responses which induce antigen-specific IFNγ^+^ T cell populations, circulating titers of anti-Ag85A IgG, and which reduce virulent *M*. *tuberculosis* burdens in murine lungs and spleens [43–46]. In a human clinical trial, use of a recombinant viral vector expressing *M*. *tuberculosis* Ag85A was well tolerated as a boosting agent to BCG and induced potent and durable T_h_1-type responses, although without evidence for increased efficacy [47–49]. Although DNA-based Ag85A primes have been shown to induce protection in experimental tuberculosis, vaccination with rBCG expressing Ag85A appears to have greater efficacy in animal models [50]. Worth noting is the fact that most tuberculosis vaccines currently in clinical trials are designed as boosting agents for BCG, suggesting that BCG is itself a viable and relevant priming agent. This supports the potential for development of improved rBCG priming vaccines not only for tuberculosis but also other important yet neglected mycobacterial diseases. Encouragingly, use of the MU-Ag85A antigen has been demonstrated to develop protection approaching that of BCG in experimental mouse models of Buruli ulcer, and served as a starting point for our recombinant BCG MU-Ag85A approach.

BCG’s extensively documented safety in immunocompetent individuals, well-established infrastructure for vaccine administration, relatively low production cost, and previously demonstrated protection make it an ideal candidate for development as an anti-Buruli ulcer vaccine vehicle. Given the prior observations that exposure to MU-Ag85A could afford some level of protection, we generated a quality-controlled, live-recombinant BCG based Buruli ulcer vaccine strain which expressed MU-Ag85A antigen. Using this strain as a prime, a subsequent administration of *M*. *smegmatis* expressing MU-Ag85A was used to examine the potential to boost primary vaccination. Two aspects of immunogenicity were examined with this vaccine: induction of antigen-specific CD4^+^ T cell proliferation and production of IFNγ-secreting cells capable of responding to MU stimulation.

Over the course of 8 weeks, BCG MU-Ag85A vaccination significantly increased levels of circulating helper T cells which recognized MU Ag85A over those levels produced by BCG. Upon analysis of MHCII tetramer staining, this proliferative effect could also be boosted for longer than the prime responses, after administration of *Msmeg* MU-Ag85A. Furthermore, by 3 weeks post-prime, significantly increased levels of antigen-specific effector and central memory CD4^+^ T cells were observed in BCG MU-Ag85A-vacinated mice versus BCG vaccination alone. Using ELISPOT analysis, mice which were primed with BCG MU-Ag85A produced significantly more IFNγ-secreting splenocytes after stimulation with MU-Ag85A peptide. Importantly, ELISPOT analysis also revealed a significant increase in the splenocyte response to heat-killed MU, suggesting that vaccination could not only increase antigen-specific recognition but also enhance T_h_1 immune responses to whole *M*. *ulcerans*.

Vaccine efficacy of BCG MU-Ag85A was also determined by investigating the potential to affect footpad swelling, bacterial burden, and overall survival of MU-challenged mice. Upon subcutaneous priming with BCG MU-Ag85A and intravenous boosting with *Msmeg* MU-Ag85A, increased time-to-onset of swelling, significantly reduced numbers of footpad acid fast bacilli, and significantly increased time of survival for MU challenged mice was observed. Previous studies have provided evidence for diminished effects of MU infection among BCG vaccinated mice, however, our data reveal that BCG overexpressing MU-Ag85A displayed enhanced protection over BCG at multiple levels of MU pathology.

Importantly, a single administration of BCG-MUAg85A alone was sufficient to significantly increase the survival of MU-challenged mice, whereas DNA-based MU-Ag85A vaccines required two prime injections and a homologous protein boost to achieve a similar level of protection observed using standard BCG [25]. Since multiple doses of BCG at 10 or 18 weeks apart were not shown to increase efficacy against experimental BU [26], we decided to use the relatively innocuous *M*. *smegmatis* to generate a boosting agent. Indeed, heterologously boosting a BCG MU-Ag85A prime with *M*. *smegmatis* overexpressing MU-Ag85A served to enhance vaccine efficacy to an even greater degree. It is interesting to note that although ELISPOT responses to the *M*. *smegmatis* were lower overall in comparison to the BCG background, boosting with *Msmeg*-MU-Ag85A did increase circulating levels of antigen-specific T cell populations. These elevated helper T cell populations remained in circulation for longer than the BCG prime alone, which may account for the enhancement of protection. Of note was the relative inhibition of bacterial replication in the footpad when comparing infected BCG or BCG MU-Ag85A-primed mice. Interestingly, a more significant 14-fold difference in bacterial load was observed 12 weeks post-infection versus 5-fold at 5 weeks, an effect which appears to synchronize with disparity in survival between those groups at similar time points. Overall, the significant protection against swelling afforded by BCG MU-Ag85A is hypothesized to be due in large part to the reduction in bacterial load, especially in light of an over 1000-fold and 100-fold reduction comparing unprimed to BCG-MUAg85A-vaccinated mice at 5 and 12 weeks post-challenge, respectively.

To our knowledge, this is the first study that has identified a Buruli ulcer vaccine candidate which has performed significantly better than standard BCG vaccination alone. As seen with numerous tuberculosis vaccine design studies however, full protection against MU-mediated pathology was not achieved by this regimen. Several factors could explain the lack of full protection, including the insufficiency of available antigens in BCG to elicit appropriate T cell populations necessary for MU containment. An alternative explanation may be associated with the large amount of evidence detailing the immunosuppressive properties of mycolactone produced by MU, whereby reduced cytokine secretion, interference with T cell signaling, and inhibition of inflammatory cell chemotaxis have all been demonstrated as functions of the mycolactone toxin [51–55]. The immunosuppressive properties of mycolactone present in the extracellular milieu may be potent enough to counteract the vaccine-induced cell-mediated responses that would have otherwise been more effective.

Use of additional animal models for vaccine efficacy testing could be more suitable in determining mechanisms of protection. The guinea pig has been well established as a model of cutaneous infections and has been used successfully in Buruli ulcer studies [37,56]. Guinea pig skin possesses more immunological and structural similarities with human skin compared to the mouse model, and may prove to be an advantageous model for future MU vaccine studies.

An alternative approach to vaccine design could be to utilize mycolactone deficient stains of MU as recombinant vaccine vehicles, which may encode antigens capable of inducing robust anti-MU immunity while lacking the virulence of wildtype MU. Such an approach has been investigated using mycolactone-negative MU5114 with limited success, but potentially more immunogenic strains or mutants of mycolactone-deficient MU could provide better protection [36]. In addition, other mycobacterial backgrounds, such as *M*. *marinum*, could present an essential antigenic profile unmet by BCG, stemming from substantial sequence homology shared with *M*. *ulcerans* [57]. Both of these approaches would undoubtedly require extensive safety profiling for use in humans, a standard already very well achieved by BCG. Despite the lack of full protection by BCG MU-Ag85A, our results lend credence to the possibility of expressing alternative MU antigens, or combinations of antigens, which may increase the protective efficacy of BCG. Others have shown that by expressing combinations of immunodominant *M*. *tuberculosis* antigens in rBCG, such as Ag85A and Ag85B together or with ESAT6 and TB10.4, bacterial burdens were further ablated over expression of single antigens [58–62]. Discovery of protective antigens specific to MU in addition to the cross-reactive antigens responsible for the baseline protection conferred by BCG will be essential to making BCG a safe and efficacious Buruli ulcer vaccine.

## Supporting Information

S1 Fig Effect of BCG dosage on protection against MU1615 challenge. C57BL/6 mice were left unprimed (dotted black) or were subcutaneously primed with 10^5^ or 10^7^ BCG. At 8 weeks post-prime, mice were challenged with 10^5^ MU1615 intradermally via the left hind leg footpad. Area of footpad swelling was measured at various time points post-challenge for 5 mice per group. Mice were sacrificed if vertical footpad swelling surpassed 4.5 mm and survival represents time to euthanasia. Asterisk indicates statistical analysis of the two BCG groups by the Mantel-Cox test. *p<0.03
(DOCX)

S2 Fig Protection assessment of *M*. *smegmatis* and *Msmeg*-MUAg85A priming against MU1615 challenge. C57BL/6 mice were left unprimed (dotted black) or were subcutaneously primed with 10^7^ *M*. *smegmatis* transformed with empty vector *Msmeg* pHA (gray) or *Msmeg* MU-Ag85A (black). At 8 weeks post-prime, mice were intravenously boosted with 10^7^ *Msmeg* expressing MU-Ag85A. Two weeks following the boost, mice were challenged with 10^5^ MU1615 intradermally via the left hind leg footpad. Area of footpad swelling was measured at various time points post-challenge for 10 mice per group. Mice were sacrificed if vertical footpad swelling surpassed 4.5 mm and survival represents time to euthanasia.
(DOCX)

S3 Fig Microscopic quantification of acid fast bacilli from footpad homogenates yield similar trends compared to CFU counting. C57BL/6 mice were subcutaneously vaccinated as previously described (unprimed; white, empty-vector BCG pHA; gray, BCG MU-Ag85A; black) and challenged with 10^5^ MU1615. At 5 weeks post-challenge, mice were euthanized and footpad homogenates were either smeared on glass slides for auramine-rhodamine staining or diluted for plating of CFU. Acid-fast bacilli (AFB) were quantified under 1000x magnification. Asterisks indicate statistical analysis by the student’s t-test (n = 16 images per group). Error bars represent standard deviation. *p<0.03, **p<0.003
(DOCX)
