# Supplementary material for: Recombinant BCG Expressing Mycobacterium ulcerans Ag85A Imparts Enhanced Protection against Experimental Buruli ulcer
Source: PLoS Negl Trop Dis. 2015 Sep 22;9(9):e0004046. doi: 10.1371/journal.pntd.0004046 (PMC4579011; doi:10.1371/journal.pntd.0004046)
Supplement: S1 Fig — C57BL/6 mice were left unprimed (dotted black) or were subcutaneously primed with 105 or 107 BCG. At 8 weeks post-prime, mice were challenged with 105 MU1615 intradermally via the left hind leg footpad. Area of footpad swelling was measured at various time points post-challenge for 5 mice per group. Mice were sacrificed if vertical footpad swelling surpassed 4.5 mm and survival represents time to euthanasia. Asterisk indicates statistical analysis of the two BCG groups by the Mantel-Cox test. *p<0.03 (DOCX) [file pntd.0004046.s001.docx]

**Supplemental Figure 1. Effect of BCG dosage on protection against MU1615 challenge.**

C57BL/6 mice were left unprimed (dotted black) or were subcutaneously primed with 10^5^ or 10^7^ BCG. At 8 weeks post-prime, mice were challenged with 10^5^ MU1615 intradermally via the left hind leg footpad. Area of footpad swelling was measured at various time points post-challenge for 5 mice per group. Mice were sacrificed if vertical footpad swelling surpassed 4.5 mm and survival represents time to euthanasia. *p<0.03
