# Supplementary material for: Recombinant BCG Expressing Mycobacterium ulcerans Ag85A Imparts Enhanced Protection against Experimental Buruli ulcer
Source: PLoS Negl Trop Dis. 2015 Sep 22;9(9):e0004046. doi: 10.1371/journal.pntd.0004046 (PMC4579011; doi:10.1371/journal.pntd.0004046)
Supplement: S2 Fig — C57BL/6 mice were left unprimed (dotted black) or were subcutaneously primed with 107 M. smegmatis transformed with empty vector Msmeg pHA (gray) or Msmeg MU-Ag85A (black). At 8 weeks post-prime, mice were intravenously boosted with 107 Msmeg expressing MU-Ag85A. Two weeks following the boost, mice were challenged with 105 MU1615 intradermally via the left hind leg footpad. Area of footpad swelling was measured at various time points post-challenge for 10 mice per group. Mice were sacrificed if vertical footpad swelling surpassed 4.5 mm and survival represents time to euthanasia. (DOCX) [file pntd.0004046.s002.docx]

**

**

**Supplemental Figure 2. Protection assessment of *M. smegmatis* and *Msmeg*-MUAg85A priming against MU1615 challenge.**

C57BL/6 mice were left unprimed (dotted black) or were subcutaneously primed with 10^7^ *M. smegmatis* transformed with empty vector *Msmeg* pHA (gray) or *Msmeg* MU-Ag85A (black). At 8 weeks post-prime, mice were intravenously boosted with 10^7^ *Msmeg* expressing MU-Ag85A. Two weeks following the boost, mice were challenged with 10^5^ MU1615 intradermally via the left hind leg footpad. Area of footpad swelling was measured at various time points post-challenge for 10 mice per group. Mice were sacrificed if vertical footpad swelling surpassed 4.5 mm and survival represents time to euthanasia.
