# Supplementary material for: Recombinant BCG Expressing Mycobacterium ulcerans Ag85A Imparts Enhanced Protection against Experimental Buruli ulcer
Source: PLoS Negl Trop Dis. 2015 Sep 22;9(9):e0004046. doi: 10.1371/journal.pntd.0004046 (PMC4579011; doi:10.1371/journal.pntd.0004046)
Supplement: S3 Fig — C57BL/6 mice were subcutaneously vaccinated as previously described (unprimed; white, empty-vector BCG pHA; gray, BCG MU-Ag85A; black) and challenged with 105 MU1615. At 5 weeks post-challenge, mice were euthanized and footpad homogenates were either smeared on glass slides for auramine-rhodamine staining or diluted for plating of CFU. Acid-fast bacilli (AFB) were quantified under 1000x magnification. Asterisks indicate statistical analysis by the student’s t-test (n = 16 images per group). Error bars represent standard deviation. *p<0.03, **p<0.003 (DOCX) [file pntd.0004046.s003.docx]

**Supplemental Figure 3. Microscopic quantification of acid fast bacilli from footpad homogenates yield similar trends compared to CFU counting.**

C57BL/6 mice were subcutaneously vaccinated as previously described (unprimed; white, empty-vector BCG pHA; gray, BCG MU-Ag85A; black) and challenged with 105 MU1615. At 5 weeks post-challenge, mice were euthanized and footpad homogenates were either smeared on glass slides for auramine-rhodamine staining or diluted for plating of CFU. Acid-fast bacilli (AFB) were quantified under 1000x magnification. Asterisks indicate statistical analysis by the student’s t-test (n=16 images per group). *p<0.03, **p<0.003
